# Proteomic Analysis of Exosome-Like Vesicles Isolated From Saliva of the Tick *Haemaphysalis longicornis*

**DOI:** 10.3389/fcimb.2020.542319

**Published:** 2020-10-22

**Authors:** Mohsin Nawaz, Muhammad Irfan Malik, Houshuang Zhang, Ibrahim A. Hassan, Jie Cao, Yongzhi Zhou, Mudassar Hameed, Zulfiqar Hussain Kuthu, Jinlin Zhou

**Affiliations:** Key Laboratory of Animal Parasitology of Ministry of Agriculture, Shanghai Veterinary Research Institute, Chinese Academy of Agricultural Sciences, Shanghai, China

**Keywords:** exosomes, extracellular vesicles, saliva, proteomics, *Haemaphysalis longicornis*

## Abstract

Extracellular vesicles (EVs), are considered as vehicles of cellular communication. Parasites usually release EVs in their excretory-secretory products to modulate host environment. However, little is known about the secretion of EVs by ticks. In this study, we show for the first time that the tick *Haemaphysalis longicornis* secretes EVs in saliva that resembles exosomes. EVs were purified from pilocarpine induced saliva of partially engorged *H. longicornis* ticks. Electron microscopy analysis revealed the presence of exosome-like vesicles with a size of 100 nm. Proteomic analysis by LC-MS/MS identified a total of 356 proteins in tick-derived EVs. Proteome data of tick-derived EVs was validated by Western blot analysis. Immunodetection of Hsp70 and GAPDH proteins indicated that the proteomics data of tick-derived EVs were highly reliable. Bioinformatics analysis (Gene Ontology) indicated association of certain biological and molecular functions with proteins which may be helpful during tick development. Likewise, KEGG database revealed involvement of vesicular proteins in proton transport, detoxification, ECM-receptor interaction, ribosome, RNA transport, ABC transporters, and oxidative phosphorylation. The results of this study provide evidence that EVs are being secreted in tick saliva and suggest that tick saliva-derived EVs could play important roles in host-parasite relationships. Moreover, EVs could be a useful tool in development of vaccines or therapeutics against ticks.

## Introduction

*Haemaphysalis longicornis*, also known as bush tick or Asian longhorned tick, belongs to the tick family Ixodidae. Although this tick is native to China, Russia, and Japan, it is now established in Pacific islands including New Zealand, Australia, and the eastern states of the USA (Heath, 2016; Berenbaum, 2018). *Haemaphysalis longicornis* is an important vector of human disease-causing agents such as thrombocytopenia syndrome virus, *Rickettsia japonica, Ehrlichia chaffeensis, Babesia microti*, and *Anaplasma bovis* (Mahara, 1997; Luo et al., 2015; Wu et al., 2017). Likewise, *H. longicornis* transmits theileriosis to cattle, thereby causing considerable blood loss and death of calves (Heath, 2016). Meanwhile, 25% reduction in dairy products has been observed in Australia and New Zealand. Parthenogenetic reproduction of *H. longicornis* allows a single female to generate progeny without mating, resulting in massive host infestations (Heath, 2016).

Extracellular vesicles (EVs) are small membrane vesicles derived from the endocytic compartment of cells. EVs have emerged as key players in intercellular communication and can be divided into exosomes, microvesicles, apoptotic bodies, and oncosomes (Devhare and Ray, 2018). Exosomes, formed by the fusion of multivesicular bodies (MVBs) were discovered in 1983 in reticulocytes (Harding et al., 2013). They were first considered as garbage bins being used by cells to discard their waste products (Johnstone, 1992). In some studies, these nano-sized vesicles were also considered as apoptotic blebs, cellular debris, or signs of cell death (Bobrie et al., 2011; Pant et al., 2012). However, the discovery of exosomes as carriers of genetic material (proteins, lipids and miRNAs) and their involvement in cellular communication has opened new horizons. Interaction of exosomes with target cells by binding with the receptors of other cells, fusion with their membranes and releasing their contents into the cytosol of target cells have made them powerful agents of cellular communication (Mathivanan et al., 2010). In addition, exosomes can even be used as vaccine candidates and biomarkers for the diagnosis and treatment of diseases (Zhang et al., 2018).

Parasites use excretory-secretory products to communicate with their host environment. Discovery of EVs within the excretory-secretory products of parasites such as *Fasciola hepatica, Echinostoma caproni, Heligmosomoides polygyrus, Dicrocoelium dendriticum, Schistosoma mansoni, Trichuris muris, Echinococcus granulosus, Leishmania amazonensis, Trichomonas vaginalis, Plasmodium vivax*, and *Trypnosoma cruzi* has gained considerable interest over the last few years (Marcilla et al., 2014; Coakley et al., 2015; Barbosa et al., 2018; Eichenberger et al., 2018; Gualdrón-López et al., 2018; Nicolao et al., 2019; RAI and Johnson, 2019). EVs can be purified form secretory products by series of steps by centrifugation, ultracentrifugation, precipitation kits, ExoChip, immunoprecipitation, acoustic nanofilter, size exclusion chromatography (SEC) column purification, and sucrose density gradient techniques (Raposo et al., 1996; Tauro et al., 2012; Kanwar et al., 2014; Lee et al., 2015; Zeringer et al., 2015; Wu et al., 2019). EVs can play a decisive role in parasite-host interactions by transferring their inner contents (virulence factors and effector molecules) from parasites to hosts (Wu et al., 2019). The content of these vesicles consists of a variety of proteins, miRNAs and lipids. Among miRNAs, high abundance of miR-71 and miR-72 has been observed in parasites suggesting their involvement in embryo development, growth, and metabolism of parasite (Chen et al., 2011; Cai et al., 2013). Furthermore, single-stranded DNA, mitochondrial DNA, double-stranded DNA and oncogene amplifications have been identified in microvesicles (Balaj et al., 2011; Thakur et al., 2014). Similarly, proteins like GAPDH, enolase, and Hsp70, usually involved in parasite survival, reproduction, and growth, have been associated within the parasite-derived EVs (Sotillo et al., 2008, 2010). In addition to miRNAs and proteins, lipids are critical components of EVs. Exosomes-like vesicles are highly enriched in an array of lipid species, including sphingomyelin, glycosphingolipids, cholesterol, and ceramide (Skotland et al., 2017; Brzozowski et al., 2018; Chen et al., 2019; Sun et al., 2019). Uptake of lipid contents by the parasites helps them to develop protective mechanisms against host immunity, support parasitic survival, and promote growth (Yesuf and Kenubih, 2019). Therefore, identification of lipid enriched EVs led us to speculate that parasite-derived EVs could provide a mechanism to modulate hosts immune responses.

EVs have been isolated from excretory-secretory products of some parasites but, to the best of our knowledge, ticks have not been studied. Here, we report for the first time that the saliva of the tick *H. longicornis* secreted exosome-like vesicles. Proteomics analysis of tick-derived EVs revealed the presence of some significant proteins such as GAPDH, heat shock proteins, thioredoxin peroxidase and proteases, which may be used by ticks in modulating host-parasite interactions.

## Methods

### Ethics Approval and Consent to Participate

All experiments carried out during the study were approved by the Institutional Animal Care and Use Committee of Shanghai Veterinary Research Institute (IACUC No: SHVRI-SOP-1104-003). Rabbits were maintained at the animal house (SHVRI) under normal conditions of regulated temperature (22°C) and light with free access to feed and water. Rabbits were kept in cages in compliance with the guidelines on the Humane Treatment of Laboratory Animals (Ministry of Science and Technology of the People's Republic of China).

### Ticks

A colony of *H. longicornis* ticks (parthenogenetic strain) was collected from Shanghai Wildlife Park, China. The tick colony was established after maintenance of three generations in the Shanghai Veterinary Research Institute, Chinese Academy of Agricultural Sciences, Shanghai, China. After feeding the ticks on New Zealand white rabbits, rearing was done in the laboratory at 25°C (92% humidity) in a dark incubator (Mulenga et al., 1999; Zhou et al., 2006). Finally, the parthenogenetic ticks were used for the collection of saliva.

### Collection of Saliva

For infestation, 40 adult ticks were attached per rabbit ear and maintained with the help of ear bags made of cotton cloth. The ear bags were held onto the rabbit ears with the help of surgical stitches and adhesive tape. After attachment, rabbits were placed in cages made of steel. A total of 55 female rabbits (4 months old) were used in this study. After feeding for 4 days, ticks were removed and saliva was collected as previously described (Patton et al., 2012). After washing with sterile distilled water, ticks were attached to glass slides with adhesive tape. Pilocarpine was injected (0.5–1 μl) posterior to fourth coxae in the region of epidermal and anal plates of the tick. Ticks were placed at 37°C in 85% humidity chamber. Saliva was collected with pipette tip after an interval of 20 min. Saliva collected from partially fed ticks was mixed with equal quantity of PBS and stored at −80°C.

### Isolation of EVS

For isolation of EVs from saliva of *H. longicornis*, a protocol described by Abdi et al. (2017) was followed with slight modifications (Supplementary Figure 1). Briefly, saliva was mixed with an equal amount of PBS and centrifuged at 2,600 g for 30 min at 4°C to remove cellular debris. Cell free medium (supernatant) was filtered through 0.22 μm filter (Merck Millipore) to remove contaminating apoptotic bodies and cell debris. Supernatant was centrifuged at 140,000 g for 3 h at 4°C in OptimaTM L-100 XP ultracentrifuge (Beckman Coulter) using an SW 60 (44.5) rotor. Supernatant was removed carefully and pellet was collected. Pellet was washed twice by re-suspending in cold PBS and centrifuging at 150,000 g for 2 h after each wash. A discontinuous gradient was prepared by diluting a stock solution of OptiPrep™ (60% w/v) with 0.25 M sucrose/6 mM EDTA, 60 mM Tris (pH 7.4). The gradient was formed by layering 40, 20, 10, and 5% gradient solutions on top of each other into 4 ml open top thin wall polyallomer (Beckman Coulter). Pellet was loaded on top of gradient and centrifuged at 250,000 g for 18 h. One milliliter fractions were collected from top of the gradient and transferred to 1.5 ml Eppendorf tubes. Weights of the tubes were measured to estimate the density of purified vesicles. A total of 5 fractions were collected, each fraction was diluted in PBS to 4 ml and centrifuged at 150,000 g for 2 h. Resulting pellet was collected and stored at −80°C. Confirmation of EVs within the saliva samples was accessed by electron microscopy.

### Electron Microscopy

The pellet was analyzed by electron microscopy at Shanghai Veterinary Research Institute, China. EV sample was fixed 1:1 with 2% glutaraldehyde. A 200 mesh copper grid with carbon-coated formvar film (Agar Scientific, Essex, UK) was incubated onto 5 μL of fixed sample for 30 min. Excess liquid was removed by blotting and grids were allowed to dry at room temperature. Grids were washed with water and stained with phosphotungstic acid for 1 min. After staining, grids were washed with ethanol (70%) followed by four washes with molecular grade water. Finally, the grids were loaded onto a sample holder of a transmission electron microscope (FEI T12 equipped with AMT XR51 CCD camera system) and exposed to 80 kV electron beam for image capture.

### SDS-PAGE Analysis and Western Blot

Purified EVs were homogenized with lysis buffer (4% SDS, 1 mM DTT, 150 mM Tris-HCl pH 8.0, protease inhibitor). After 3 min incubation in boiling water, the homogenate was sonicated on ice. The crude extract was then incubated in boiling water again and clarified by centrifugation at 16,000 × g at 25°C for 10 min. The concentration of proteins was determined using a Micro BCA Protein Assay Kit (Thermo Fisher Scientific, Rockford, IL, USA) following the manufacturer's specifications and using BSA (Thermo Fisher Scientific) as a standard. Five micrograms of protein per lane were processed by SDS-PAGE (12% polyacrylamide linear gradient gels; Bio-Rad Laboratories, Hercules, CA, USA) and stained with Coomassie Brilliant blue R-250. Gels were scanned using a Bio-Rad Molecular Imager FX system (Bio-Rad Laboratories).

For western blot analysis, 7 μg of proteins were subjected to 12% SDS-PAGE. It was followed by the transfer of proteins to polyvinylidene fluoride (PVDF) membranes. The membranes were blocked with 5% skim milk diluted in PBS/0.05% Tween (PBST) for 2 h at 37°C. Membranes were washed three times (5 min/washing) with PBST. Blots were incubated overnight at 4°C in rabbit anti-Hsp70 antibody diluted to 1:1,000 in PBST (cat. no. ab79852; Abcam) and rabbit anti-GAPDH antibody diluted to 1:1,000 in PBST (cat. no. ab37168; Abcam). Blotted membranes were washed three times with PBST (10 min/washing) and incubated in the presence of goat anti-rabbit IgG antibody (horseradish peroxidase-conjugated; dilution, 1:2,000; Bethyl Laboratories, Inc., USA) for 1 h at 37°C. Washing with PBST (10 min/washing) was done before visualization of bands. Protein signals were detected with an Enhanced Chemiluminescent Substrate Reagent Kit (NCM Biotech, Sunzhou, China) and were visualized under a Tanon-5200 Chemiluminescent Imaging System (Tanon Science and Technology, Shanghai, China).

### Liquid Chromatography Mass Spectrometry (LC-MS/MS)

Purified samples (EVs) dissolved in PBS were diluted in 30 μl SDT buffer (4% SDS, 100 mM DTT, 150 mM Tris-HCl pH 8.0) and boiled for 5 min. The detergents (DTT and other low-molecular-weight components) were removed using 200 μl UA buffer (8 M Urea, 150 mM Tris-HCl pH 8.0) by repeated centrifugation (14,000 × g for 15 min). After centrifugation, the concentrates were mixed with iodoacetamide (IAA, 50 mM IAA in UA) and incubated in darkness for 30 min at room temperature. After 15 min centrifugation, filters were washed three times with 100 μL UA buffer and then 100 μL of dissolution buffer (50 mM triethylammonium bicarbonate, pH 8.5) twice. The tryptic peptides resulting from the digestion were extracted with 0.1% formic acid in 60% acetonitrile. Protein suspension was digested with 2 μg trypsin (Promega, Madison, USA) in 40 μL 25 mM NH4HCO3 overnight at 37°C. The extracts were pooled and completely dried using a vacuum centrifuge.

For protein identification, liquid chromatography-mass spectroscopy assay (LC-MS/MS, Thermo Fisher Scientific) was performed using Q Exactive™ Plus Hybrid Quadrupole-Orbitrap™ Mass Spectrometer coupled on Easy NLC system 1000 (Thermo Fisher Scientific). Five micrograms of proteins were used by LC-MS/MS. Trypsin digested peptides were desalted on Zorbax 300SB-C18 peptide traps (Agilent Technologies, Wilmington, DE, USA) and separated on a C18-reversed phase column (0.15 × 150 mm, Column Technology Inc., Fremont, CA, USA). Mobile phases A (0.1% formic acid in HPLC-grade water) and B (0.1% formic acid in 84% acetonitrile) were delivered using an Easy nLC system (Thermo Fisher Scientific) with a linear gradient of 4–50% B (50 min), 50–100% B (4 min), and 100% B (6 min) at a flow rate of 250 nl/min. A data-dependent method, based on 10 most abundant precursor ions for HCD fragmentation was used to acquire mass spectrometry data. For survey scans (m/z 300–1,800), the target value was determined based on predictive Automatic Gain Control at a resolution of 70,000 at m/z 200 and dynamic exclusion duration of 25 s. The resolution set for the HCD spectra was 17,500 at m/z 200. Normalized collision energy was 27 eV and the under-fill ratio was set as 0.1%.

For in gel protein identification, Ettan™ MDLC controlled by UNICORN™ software (GE Healthcare), was used for desalting and separation of peptides. Peptide mixtures were desalted on RP trap columns and then separated on a C18-reversed phase column. Mobile phase A (0.1% formic acid in HPLC-grade water) and mobile phase B (0.1% formic acid in 84% acetonitrile) were selected. Tryptic peptide mixtures were loaded onto the columns, and separation was done at a flow rate of 2 μL/min by using the linear gradient buffer B described above. LTQ Velos (Thermo Scientific) equipped with a micro-spray interface was connected to the LC setup for eluted peptides detection. Data-dependent MS/MS spectra were obtained simultaneously. Each scan cycle consisted of one full scan mass spectrum (m/z 300–1,800) followed by 20 MS/MS events of the most intense ions with the following dynamic exclusion settings: repeat count 2, repeat duration 30 s, exclusion duration 90 s.

### Database Searching and Protein Identification

MaxQuant software (version 1.3.0.5.) suite was used to process Raw MS files. Peak list files were analyzed using Mascot search algorithm (Matrix Science Matrix Science, London, UK; version 2.2) against the UniProtKB Ixodoidea database (downloaded at July 06, 2018, with 190,922 entries) containing both forward and reverse protein sequences. The search parameters were: trypsin enzyme; two missed cleavages; a fragment ion mass tolerance of 0.10 Da; mascot score >20 and peptide tolerance of 20 ppm. Carbamidomethyl of cysteine was specified in Mascot as a fixed modification whereas oxidation of methionine was specified as variable modification. Proteins with at least two peptide (false discovery rate < 0.01) uniquely assigned to the respective sequence were considered.

Gene ontology (GO) is a universally acknowledged functional enrichment database and is generally used to search for enriched GO terms. Differently expressed proteins were classified into GO annotations according to molecular function, biological process, and cellular component. Additionally, KEGG database was mapped to analyse the pathways of the proteins involved.

### Statistical Analysis

Right-tailed Fisher's exact test was used to access Gene enrichment of three ontologies (biological processes, cell components, and molecular functions) and KEGG pathway enrichment analysis. GO analyses were performed using DAVID (v6.8; https://david.ncifcrf.gov/) and Cytoscape online software (https://cytoscape.org/) (Shannon et al., 2003; Sherman and Lempicki, 2009; Xing et al., 2016). Unpaired *t*-tests were used to perform all statistical analyses. *P* < 0.05 was considered to indicate significant differences. All assays were performed in three replicates.

## Results

### *Haemaphysalis longicornis* Saliva Produce Exosome-Like Vesicles

**Figure 1:**
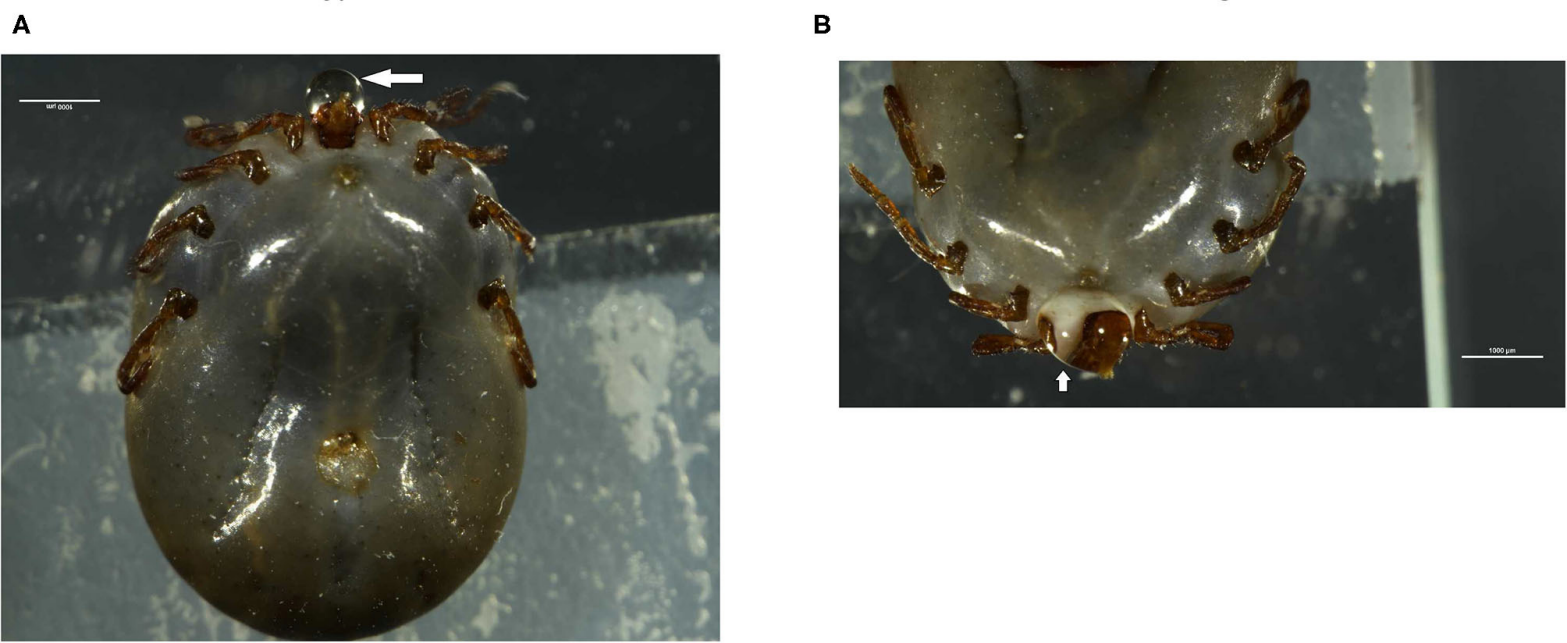
Collection of saliva from partially engorged *H. longicornis* ticks. **(A,B)** Ticks were injected with pilocarpine and 0.5–1 μl of saliva was collected. Arrows indicate the saliva secreted by ticks.

**Figure 2:**
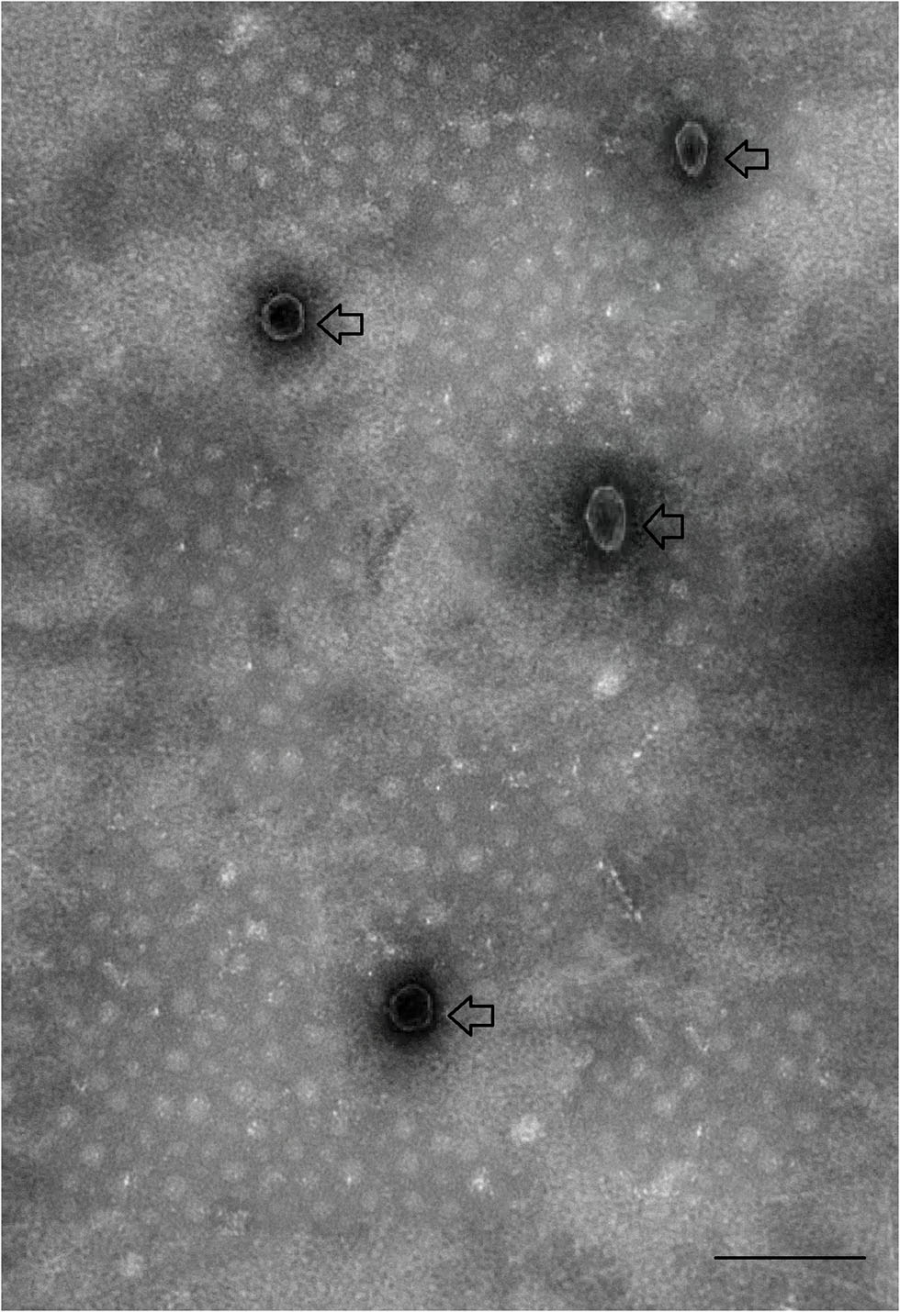
Transmission electron micrograph of exosomes-like vesicles derived from tick saliva. Arrows indicate tick-derived EVs. Scale-bar: 100 nm.

Saliva was collected from partially fed (4 days post-feeding) *H. longicornis* after injecting with pilocarpine as shown in Figures 1A,B. A total of 2,500 μl of saliva was collected from ~4,000 ticks. EVs, isolated from saliva were observed by electron microscopy. Vesicles appeared within saliva were having a size of 100 nm, which is usually the range of exosomes (Figure 2). Exosomes appeared as typical spherical structures released into the saliva, as previously observed in the case of other parasites (Samoil et al., 2018). To ascertain whether these vesicles were also present in supernatants after ultracentrifugation, we next carried out purification of these structures from supernatants using TEM to visualize them. However, structures resembling EVs were not found in supernatant.

### Tick EVs Contain Proteins

**Figure 3:**
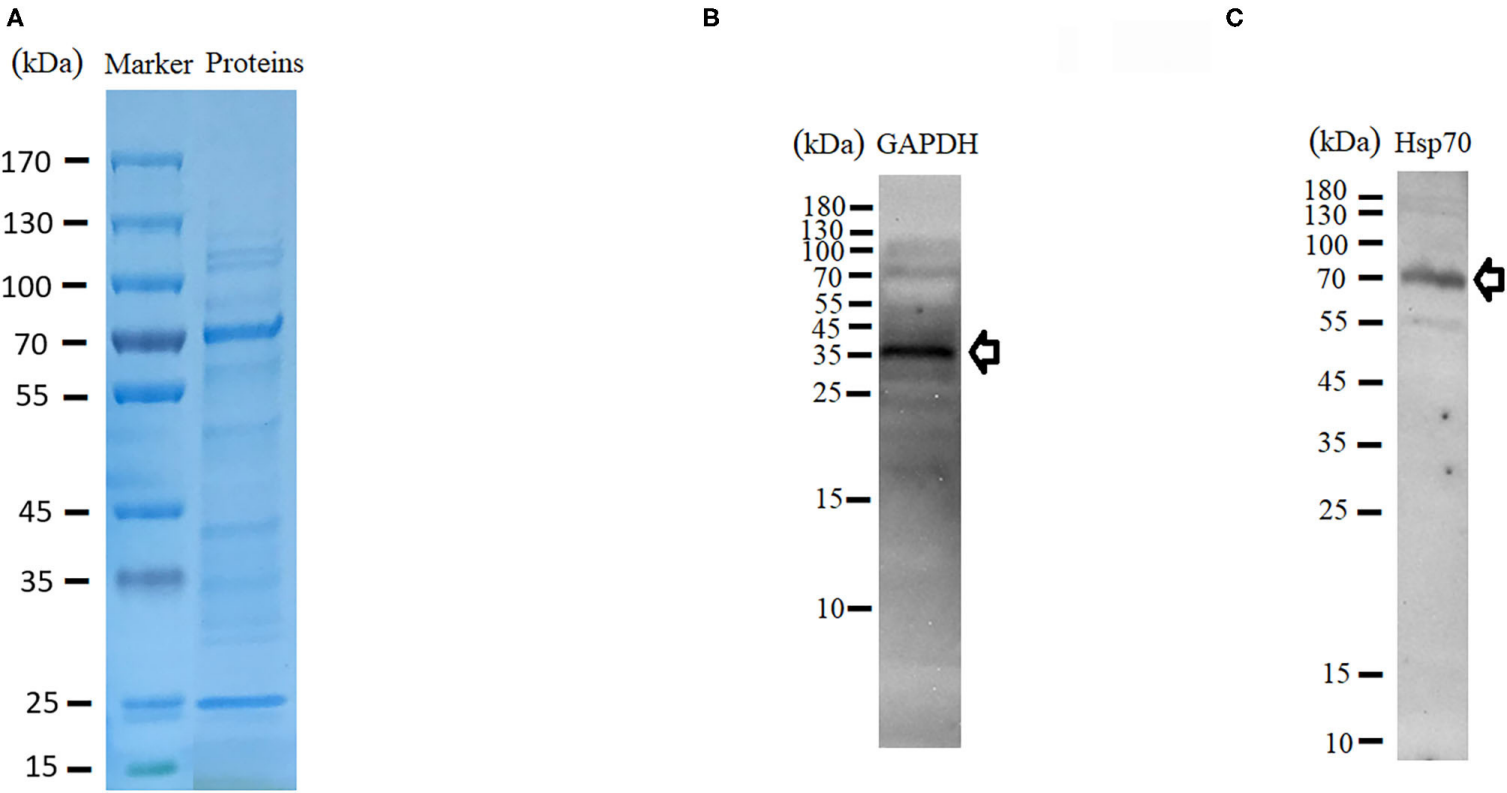
Characterization of protein contents of tick-derived EVs. **(A)** SDS-PAGE and Coomassie blue stained. **(B,C)** Validation of GAPDH and Hsp70 through Western blot.

Proteomics (LC-MS/MS) was carried out to identify proteins present within purified EVs. SDS-PAGE followed by Coomassie blue staining indicated an enrichment of specific proteins (Figure 3A). Protein identification using MASCOT confirmed the presence of 356 proteins (Supplementary Table 1). Most of the proteins like nuclear proteins (elongation factors and histones), cytoskeletal proteins (actin, tubulin), and stress-related proteins (HSPs) have been identified in previous studies. Proteins have been classified in groups based on function and/or protein families (Table 1). In addition, EVs isolated from tick saliva were found to be enriched with host proteins. MASCOT searches identified the presence of 225 host proteins mainly corresponding to immunoglobulins, histones as well as metabolic proteins (Supplementary Table 2). However, deep analysis revealed slight differences (peptide counts and cover percent) between host proteins and vesicular proteins, e.g., peptide counts for GAPDH from host proteins were 3 while only 2 counts were observed for EVs-derived GAPDH. Similar differences were also observed in other proteins like histones. The presence of host-derived proteins suggests that tick EVs are involved in host-parasite relationships.

**Table 1:** Proteins identified in tick-derived EVs by LC–MS/MS.

**Uniprot ID**	**Description**	**Uniprot ID**	**Description**
**Protein synthesis machinery**			
A0A2R5LAZ1	Putative 40s ribosomal protein s3	A0A2R5LH55	Ribosomal protein s18
A0A131Z199	E3 ubiquitin-protein ligase TRIP12	A0A131ZAN1	Small subunit ribosomal protein S27Ae
A0A1E1XMP5	Putative e3 ubiquitin-protein ligase herc2	A0A023FFH9	Mitochondrial ribosomal protein mrp-s35
A0A1E1XUQ4	RBR-type E3 ubiquitin transferase	A0A023FHE7	Ribosomal protein l34
B7PFT8	U4/U6 small nuclear ribonucleoprotein Prp4	A0A131YU15	Small subunit ribosomal protein S7
A0A2R5LAX2	60s ribosomal protein l17	A0A1E1XHY1	Ribosomal protein l3
A0A2R5LH55	Ribosomal protein s18	A0A1Z5LGI7	60s ribosomal protein L10
**Heat shock proteins**			
A0A023FJK7	Putative heat shock protein	B7Q057	HEAT repeat-containing protein
A0A023GP15	Putative heat shock protein	M9WB33	Heat shock protein 90
A0A131YY80	Heat shock 70 kDa protein		
**Translation regulation**			
A0A147BCU5	Translation initiation factor 5b eif-5b	A0A293N8J1	Elongation factor 1-alpha
A0A1E1XCT0_	Translation initiation factor eif-2b subunit delta-like isoform 1	A0A147BP71	Elongation of very long chain fatty acids protein
V5I557	Negative regulation of translation involved in protein silencing by mirna		
**Nuclear regulation**			
A0A023GER7	Histone H2A	A0A2R5L410	Endothelial zinc finger protein induced by tumor necrosis factor alpha
A0A131XUE0	Component of histone deacetyl	V5IF43	Lamin
A0A240EVT3	Histone H3	A0A2R5LHU4	Transcription factor a mitochondrial
A0A0C9RRX6	Histone H2B	A0A131YIB9	Transcriptional regulator ATRX
A0A023FTG4	Histone H4	A0A293LF12	Zinc finger protein
**Signal transduction**			
A0A293MX67	Ras-related protein	V5HIK9	Ras-related protein rab-11a
**Glycine rich**			
A0A023FQ63	Glycine-rich cell wall structural protein 1.8		
**Lipocalins (4)**			
A0A023GD09	Lipocalin-2 1	A0A131Z256	Lipocalin
A0A131YQ62	Lipocalin	A0A147BW77	salivary lipocalin
**Cement (2)**			
A0JC33	Cement-like antigen	A0JC35	Cement-like antigen
**Cytoskeletal (11)**			
L7LY53	Myosin light chain binding protein	A0A1E1XF25	Microtubule-actin cross-linking factor 1-like protein
A0A147BK70	Myosin-16	A0A109QJ05	Tropomyosin
A0A1E1X2T4	Myosin-2 heavy chain	A0A147BWI1	Tropomyosin-2 isoform 4
L7LY53	Myosin light chain binding protein	A0A2R5L9T9	rhoa gtpase effector dia/diaphanous
A0A1E1X349	Actin related protein 1	A0A293M7V9	Tubulin alpha chain
A0A023FMG5	Actin-binding cytoskeleton protein filamin		
**Serpins (3)**			
A0A090X8Z2	Serine protease inhibitor	B7Q8M7	Serpin-2 precursor
A0A131YIT6	Pancreatic trypsin inhibitor		
**Enzymes (68)**			
*A0A1Z5L0Z6*	*Enoyl-CoA hydratase*	A0A023GFD5	Carboxypeptidase
B5M758	3-hydroxyacyl-coa dehyrogenase	A0A023GK80	cd73 ecto-5′-nucleotidase
A0A023G073	2-oxoglutarate dehydrogenase e1 subunit	A0A0C9S1T0	gdp-l-fucose synthetase
A0A0K8RQB8	Dihydrolipoyl dehydrogenase	A0A0K8R7B1	Glutathione s-transferase 1
A0A131XU72	Hydroxysteroid dehydrogenase-like protein 2	A0A1E1XJE7	Sumo1/sentrin specific peptidase 6a
A0A131YNN3	15-hydroxyprostaglandin dehydrogenase (NAD)	A0A293LZ27	GDP-fucose protein O-fucosyltransferase 1
A0A1Z5KY25	Malate dehydrogenase	A0A2R5LGC2	Glyoxalase
A0A293MCU1	Glutaryl-CoA dehydrogenase	A0A2R5LGN0	Dual specificity phosphatase
A0A0K8RBY4	Peptidyl-prolyl cis-trans isomerase	A0A2R5LJB8	rna polymerase-associated protein ctr9
A0A131YPA8	Peptidylprolyl isomerase	A0A2R5LK45	Argininosuccinate synthase
A0A147BS85	Putative prolyl 4-hydroxylase alpha subunit	A0A2R5LN87	ran gtpase-activating protein
A0A2R5LKC5	Putative cytosol aminopeptidase	B7P6A7	ATP synthase subunit alpha
A0A023FM97	Aminopeptidase	B7PB22	Calcium-dependent cysteine protease
A0A131XGJ9	Putative ftsj-like rna methyltransferase	B7Q0Z1	GTPase
A0A131XZ89	Alpha-mannosidase	B7Q2U6	Nicotinamide N-methyltransferase
A0A131YR95	Amidase	A0A131ZD23	Reprolysin
A0A147B8Y3	Thioredoxin peroxidase	A0A224YV60	O-phosphoseryl-tRNA(Sec) kinase
A0A147BSL3	Putative thymidylate synthase	B7PXE6	Phosphatidylinositol 4 kinase
A0A1E1X8M7	ATP synthase subunit beta	B7Q6K8	Phosphatidylinositol 3-kinase catalytic subunit
A0A293N4N6	Catalase	A0A1E1XUQ4	RBR-type E3 ubiquitin transferase
A0A2R5L8K4	Putative abc transporter atp-binding protein/permease	Q86GZ5	Midgut cysteine proteinase 2
A0A2R5LGC2	Putative glyoxalase	A0A1Z5L983	Sulfotransferase
A0A2R5LGN0	Putative dual specificity phosphatase	A0A224YIB1	ATP-dependent RNA helicase DHX33
A0A2R5LK45	Putative argininosuccinate synthase	B7Q3E5	Citrate lyase beta chain
A0A2R5LKC5	Putative cytosol aminopeptidase	A0A224Z2B8	Malonyl coaacp transacylase
B7PB22	Calcium-dependent cysteine protease	A0A293MCU1	Glutaryl-CoA dehydrogenase
V5H108	DNA-directed RNA polymerase III subunit	A0A293MQS4	NADPH-dependent diflavin oxidoreductase 1
B7Q0Z1	GTPase, putative	L7MCS1	Putative mrna splicing factor atp-dependent rna helicase
B7Q2U6	Nicotinamide N-methyltransferase, putative	Q4R1A6	Metalloprotease
B7QCU5	Sulfotransferase, putative	*Q86GZ5*	*Midgut cysteine proteinase 2*
A0A131YGI5	RHIAP Angiotensin-converting enzyme	V5HY02	Putative tick metalloprotease
L7S6B3	Glutathione peroxidase	V5HZQ9	Putative endoribonuclease dcr-1
A0A023FMK0	Aspartate aminotransferase	A0A023FVG3	Angiotensin-converting enzyme
G8C7A0	Lysosomal acid phosphatase	A0A293LC34	Farnesyltransferase alpha subunit
A0A2P1DPZ4	Glyceraldehyde-3-phosphate dehydrogenase		
**Secreted proteins (24)**			
A0A023G1E9	Putative secreted protein	A0A023FEZ7	Putative secreted mucin
A0A023G2A9	Putative secreted protein	A0A1E1XQT0	5′-nucleotidase
A0A023GE86	Putative secreted protein	A0A1E1X7A9	cd73 ecto-5′-nucleotidase
A0A090X9H5	Putative secreted protein	A0A023GD61	glycosyl hydrolase family 38
A0A090XCY0	Putative secreted protein	A0A0K8RNX7	m13 family peptidase
A0A0K8R869	Putative secreted protein	A0A1Z5LIJ2	Thioredoxin-dependent peroxide reductase mitochondrial
A0A131Y3N9	Putative secreted protein	A0A2R5LK45	Argininosuccinate synthase
A0A1E1WY51	Putative conserved secreted protein	A0A147BG74	Acyltransferase required for palmitoylation of hedgehog hh family of secreted signaling
A0A1E1X1V4	Putative secreted protein	A0A023FL62	f0f1-type atp synthase alpha subunit
F0J8F4	Hypothetical secreted protein 1752	V5ICE2	Putative secreted protein
V5HCM2	Putative secreted protein	A0A131XF49	Putative secreted salivary gland peptide
V5I529	Putative secreted protein	A0A131XLI6	Putative secreted metalloprotease
**Transporters (25)**			
M5AYG7	Ferritin	A0A131YI50	Vitellogenin-3
Q6WNX5	Ferritin	A0A023FUV2	Vitellogenin-2
E1CAX9	Vitellogenin-1	A0A023FYX2	Vitellogenin-2
B1B544	Vitellogenin-2	A0A023GCA7	Vitellogenin-2
E1CAY0	Vitellogenin-3	A0A2R5L9R5	Vitellogenin-1
G9M4L6	Vitellogenin-B	A0A1E1X1H2	Vitellogenin-c
A0A023GME3	Vitellogenin-1	Q19V51	Hemelipoglycoprotein
A0A023GMC7	Vitellogenin-1	B5ABL8	Hemelipoglycoprotein 2
V5H7G7	Vitellogenin-2	A0A023FKG8	Lipid exporter abca1
A0A023GNW9	Vitellogenin-2	A0A023FWZ5	Nuclear pore complex protein nup85
A0A034WXH7	Vitellogenin 4	A0A1Z5L0J3	Aquaporin
A0A023GPB4	Vitellogenin-2	A0A224Z507	Nuclear pore complex protein Nup188
A0A2R5L4N6	Spatacsin		
**Immunity related (5)**			
A0A1E1XEL3	Alpha-macroglobulin	G3BJU6	Immunogenic protein
A0A023FNM2	Alpha-2-macroglobulin-like protein	A0A0K8R7R0	Ixodes 26 kDa salivary protein
A0A224YHA0	Alpha-2-macroglobulin splice variant 1		

*Proteins were classified in groups based on function and/or protein families*.

In order to validate proteome data of tick-derived EVs, the supernatant collected during centrifugation was also examined for the presence of proteins within it. LC-MS/MS identified 490 proteins within supernatant, which may be considered as tick saliva proteins (Supplementary Table 3). Presence of these proteins within supernatant suggests that the proteins within pellet are specific to tick EVs. Likewise, western blot analysis was carried out to get further confirmation. For this purpose, HSP70 and GAPDH proteins were selected. Immunodetection of proteins resulted in thick bands of 36 and 70 kDa. The predicted size of GAPDH is 35.6–36 kDa, which matches the observed band (Figure 3B). Similarly, the band observed at 70 kDa matches to Hsp70 (Figure 3C). Validation of the proteins by Western blot indicated that the proteomics data of tick-derived EVs were highly reliable.

**Figure 4:**
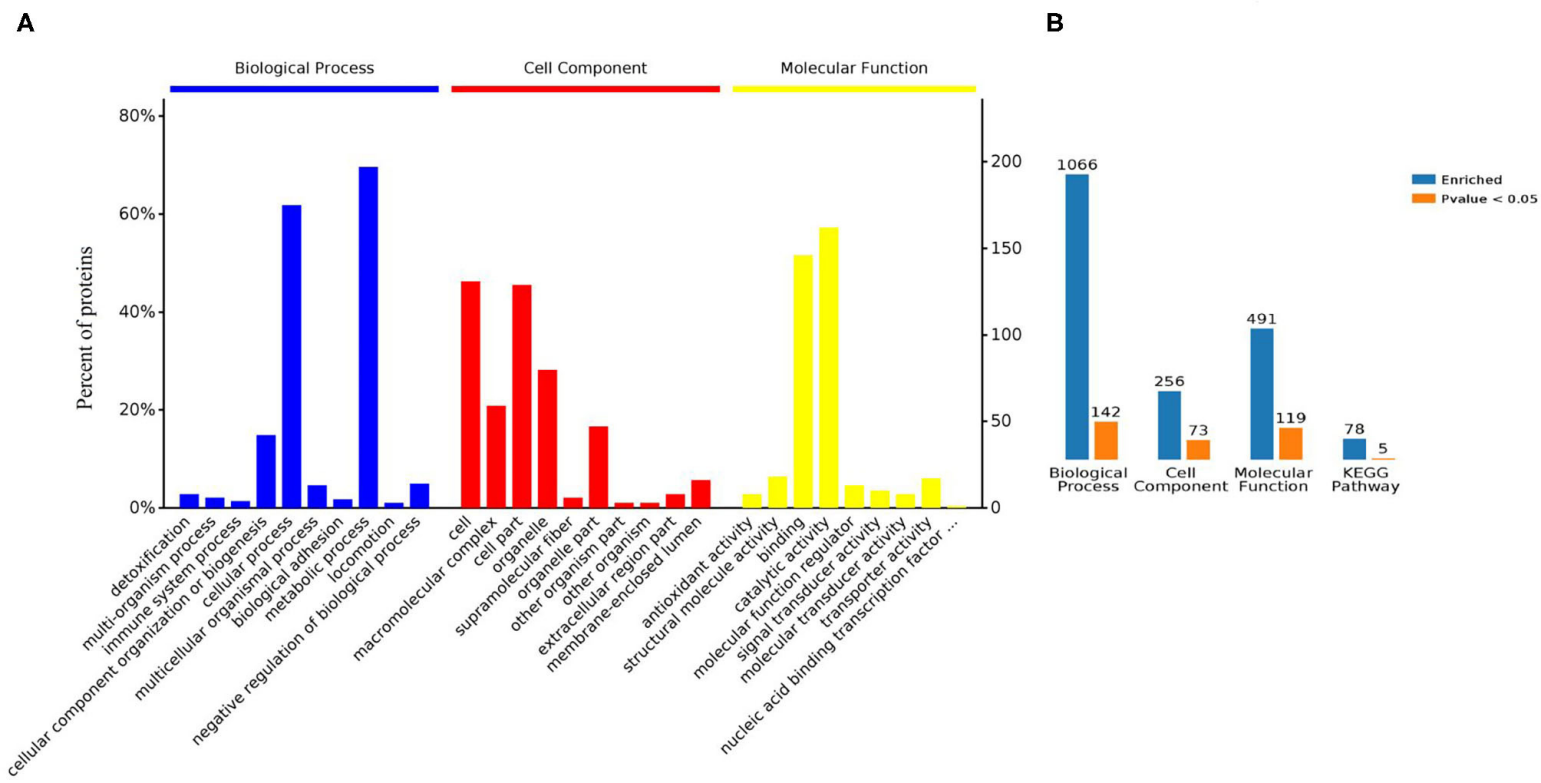
Bioinformatic analyses of the proteins derived from tick EVs. **(A)** Enrichment analysis by biological process, cell component, and molecular function. **(B)** Enriched GO terms and KEGG pathways analysis of differently expressed proteins: blue: enriched; orange: significantly enriched.

**Figure 5:**
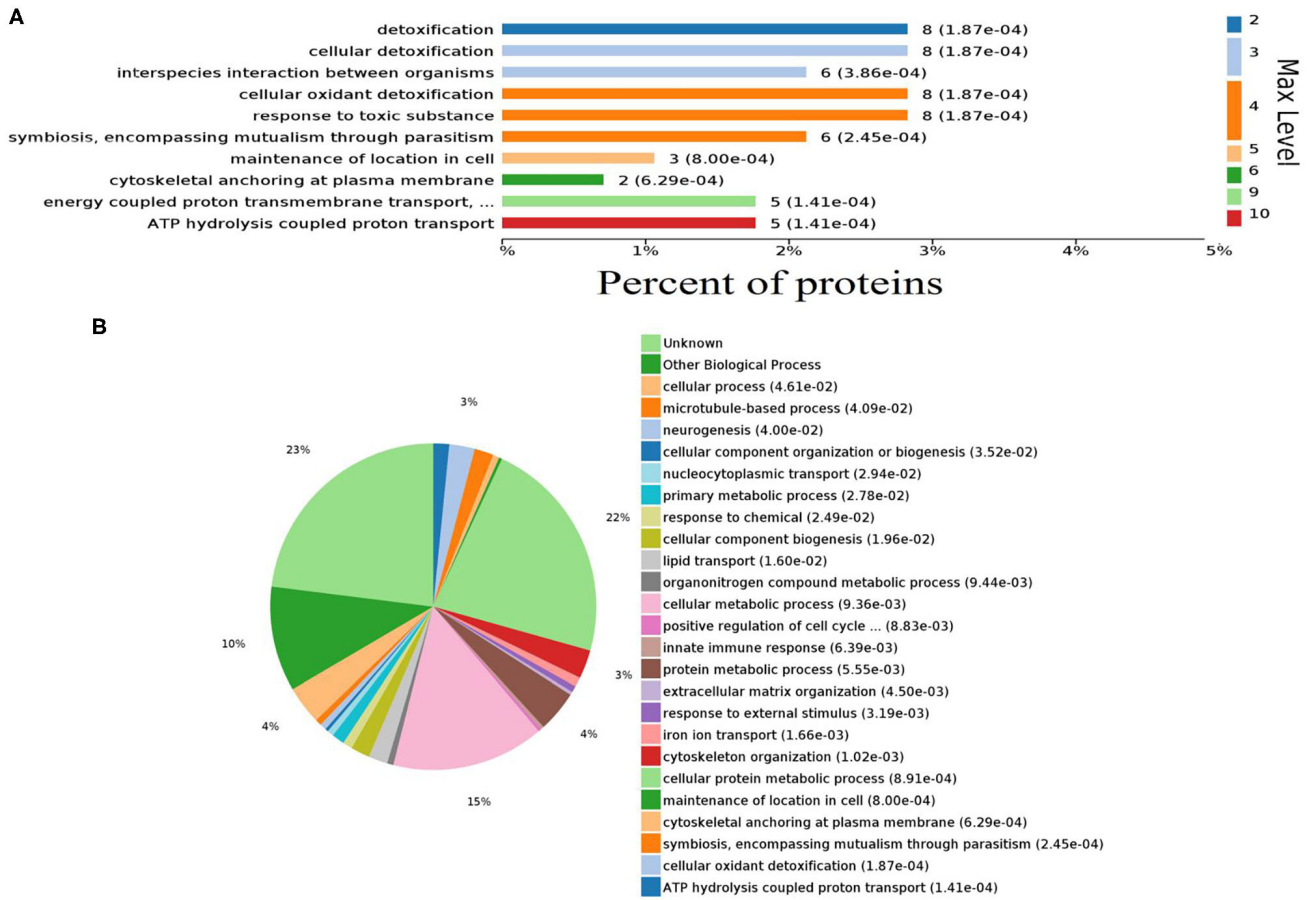
Abundantly represented gene ontology biological process terms. **(A)** Bar chart showing the percentage of proteins involved in biological process. **(B)** Pie chart distribution of enriched biological processes.

**Figure 6:**
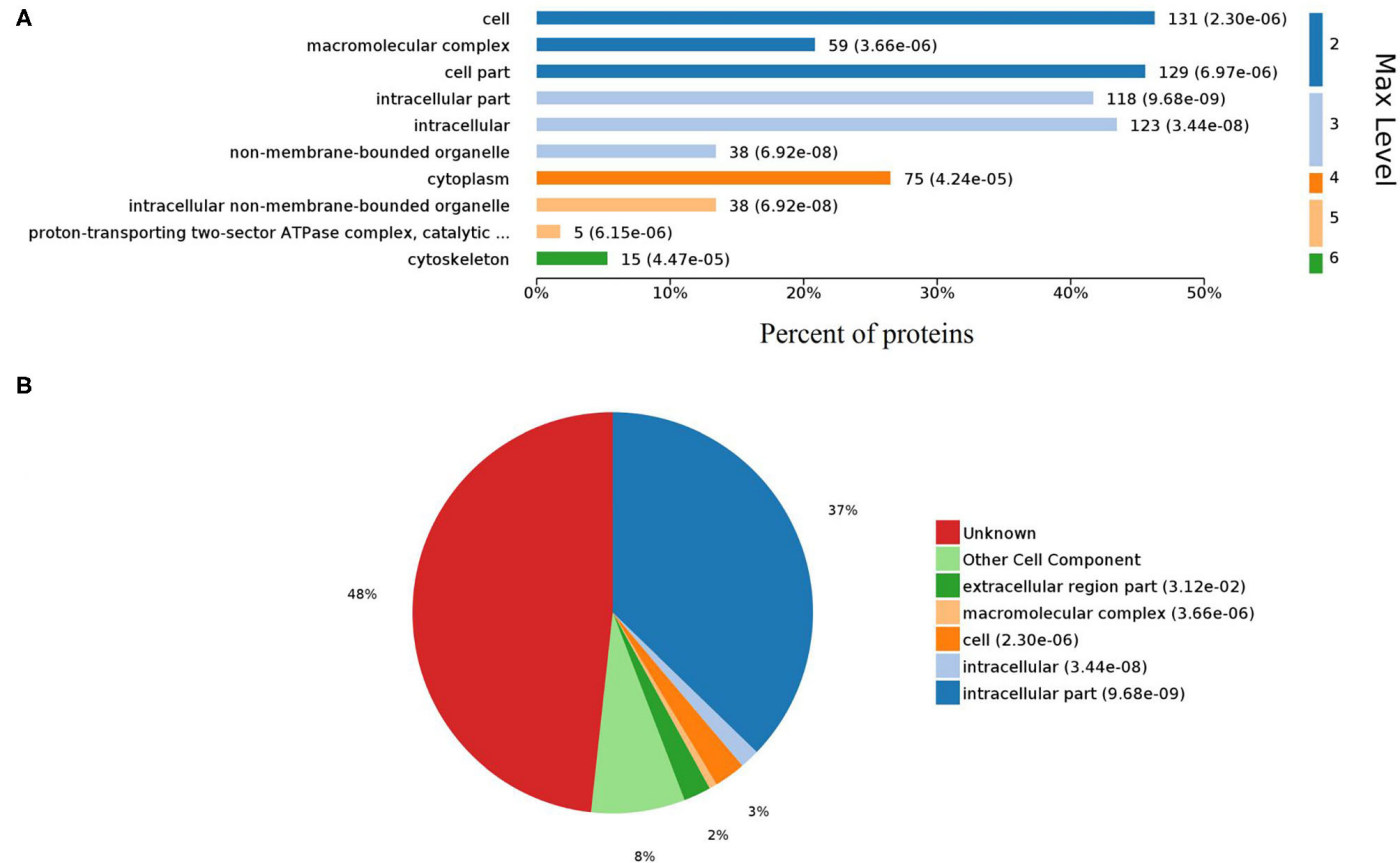
Gene Ontology analysis of cellular components. **(A)** Bar chart representing the levels of cell components. **(B)** Pie chart showing the classification of cell components.

**Figure 7:**
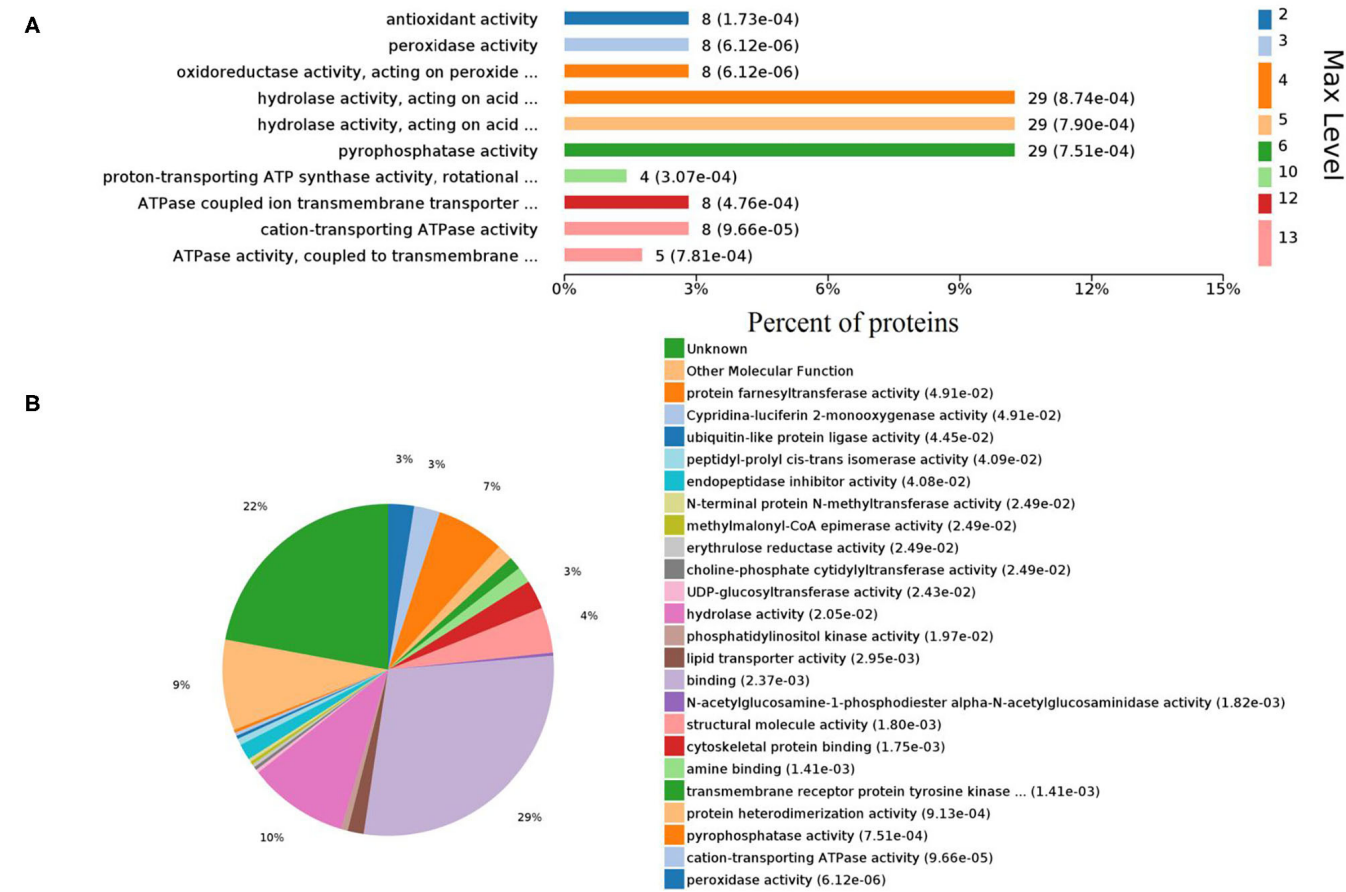
Enriched GO terms of molecular functions. **(A)** Different levels of molecular functions. **(B)** Pie chart denoting the distribution summary of molecular functions.

Gene ontology (GO) analysis of differentially expressed proteins showed significant enrichment for 142 proteins associated with “Biological process,” 73 associated with “Cellular components,” and 143 associated with “Molecular function” (Figures 4A,B, Supplementary Table 4). In terms of biological process, the *P*-value (*P* < 0.05) indicated that ATP hydrolysis coupled proton transport, energy coupled proton transmembrane transport, cellular oxidant detoxification, detoxification, and cellular detoxification were more significant (Figures 5A,B, Supplementary Figure 2A). Analysis of “Cellular components” indicated that most of the proteins were associated with the “cell” category. In addition, further subdivisions revealed that differentially expressed proteins were involved in intracellular parts, cytoplasm, proton-transporting two-sector ATPase complex, and cytoskeleton (Figures 6A,B, Supplementary Figure 2B). Significant “Molecular functions” associated with vesicular proteins were antioxidant activity, peroxidase activity, hydrolase activity, pyrophosphatase activity, and ATPase activity (Figures 7A,B, Supplementary Figure 2C).

**Figure 8:**
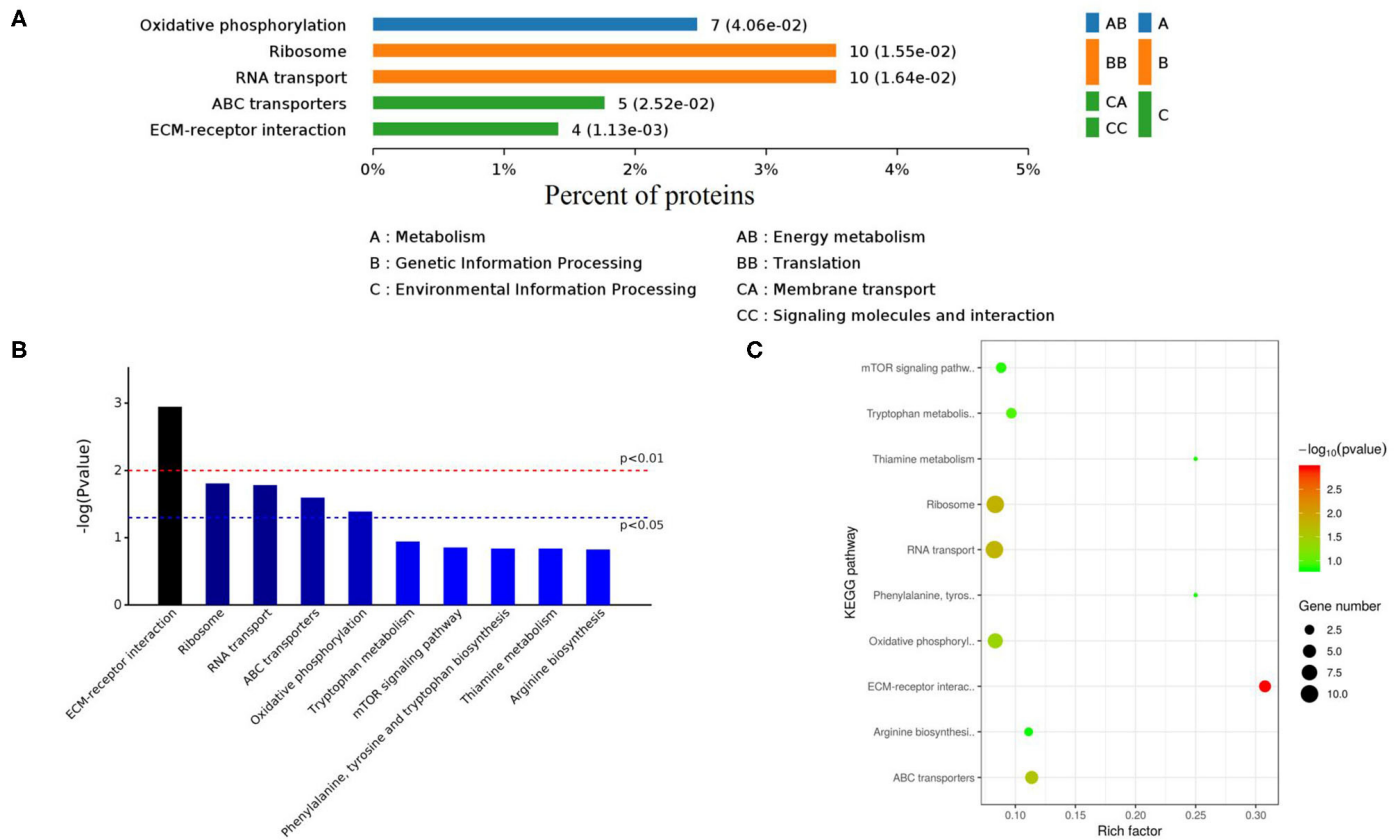
KEGG enrichment pathways of differently expressed proteins. **(A)** Classes of enriched KEGG pathways. **(B)** Distribution of KEGG pathways (Top 10). **(C)** Scattered plot of differently expressed proteins. X-axis indicates the rich factor; Y-axis indicates the name of KEGG pathway. The dot size means the protein number and dot color indicates the *q*-value.

The KEGG database is a collection of various pathways, representing the molecular interactions and reaction networks. To identify the pathways involved, we mapped the KEGG database and found that identified proteins were enriched in 78 pathways (Figure 8A, Supplementary Table 5). Further analysis of the *P*-values (*P* < 0.05: Figures 8B,C) revealed that differentially expressed proteins were mainly involved in ECM-receptor interaction, ribosome, RNA transport, ABC transporters, and oxidative phosphorylation.

## Discussion

EVs are known to transfer intracellular information from one cell or tissue to other (Nawaz et al., 2019; Wu et al., 2019). The information (proteins, miRNAs) transferred by EVs are thought to mediate cellular activity and pathways in recipient cells (Zhu et al., 2016). Recent studies revealed that parasites such as *Schistosoma japonicum, Leshmania infantum, Toxoplasma gondii*, and *Fasciola hepatica* release EVs in their excretory-secretory products (Cwiklinski et al., 2015; Zhu et al., 2016; Li et al., 2018; Marshall et al., 2018). In the context of ticks, Zhou et al. (2018) demonstrated that tick embryonic cell line *Ixodes scapularis* ISE6 is also capable of secreting extracellular vesicles including exosomes. However, it remained unknown whether ticks such as *H. longicornis*, a major agent causing severe pathology of theileriosis, could secrete vesicles in saliva as well. Here, we isolated vesicles from saliva of *H. longicornis*. Transmission electron microscopy was carried out to examine EVs. Consistent with previous reports, we demonstrated that *H. longicornis*-derived EVs are similar in size (100 nm) and shape to other parasite-derived exosomes.

LC-MS/MS showed a wide variety of proteins within tick-derived EVs. The major protein groups included structural proteins, metabolic proteins, nuclear proteins, transporters, enzymes, and some proteins for which no homologs (Table 1). Structural/cytoskeletal proteins such as myosin, tropomyosin, actin, and tubulin alpha chain found in our study have been recorded with high scores previously in parasite-derived EVs such as *B. malayi, S. japonicum* and *L. infantum* (Atayde et al., 2015; Zamanian et al., 2015; Zhu et al., 2016). Actin, representing 1.1% of proteins identified, has been known to play immunogenic role in *Echinostoma caproni* (Sotillo et al., 2010). Likewise, myosin proteins (1.7%) localized beneath the plasma membrane exhibit biophysical properties required to generate fast movements in parasites such as *T. gondii* and other apicomplexans (Sibley et al., 1998; Herm-Götz et al., 2002). The presence of structural proteins suggests that they may be associated with the production of vesicles (Wubbolts et al., 2003).

The vitellogenin group of proteins, representing 4.5% of proteins identified in our study, have not been previously identified in EVs of other parasites. Vitellogenin (Vg), also considered as female-specific protein, is synthesized as a high molecular-mass precursor in ovaries, gut, and fat body of ixodid ticks (Rosell and Coons, 1992; Thompson et al., 2007; Boldbaatar et al., 2010). After its release into the haemolymph, Vg is taken up by oocytes through receptor mediated endocytosis, and is then accumulated in yolk granules.Vg is considered as a source for embryo development and egg formation during tick reproduction (Xavier et al., 2018). Antioxidant property of Vg, diminishing heme-induced lipid peroxidation has been reported. Importantly, silencing of Vg in ticks feeding on sheep resulted in reduced engorgement and oviposition rates (Esteves et al., 2017). Likewise, the protein ferritin identified within the tick EVs, has been known to play crucial role in blood-feeding and reproduction in *H. longicornis* and *Ixodes ricinus* (Hajdusek et al., 2009; Galay et al., 2013). Moreover, insect ferritin was also implicated in iron transport, immune response and oxidative stress (Orino et al., 2001; Ong et al., 2006). As these proteins have been identified in saliva of ticks, therefore, it has been believed that tick saliva is beneficial for reproduction and blood-feeding of ticks. However, the association of these particular proteins with tick EVs still requires further confirmation.

Some other proteins like heat-shock proteins (HSPs), thioredoxin peroxidase, metalloprotease, glyceraldehyde-3-phosphate dehydrogenase, and glutathione S-transferase have also been identified in the context of parasite-derived EVs. HSPs play key roles in differentiation, adaptation, and protection of parasites from killing mechanisms of hosts such as low pH and reactive oxygen metabolites (Johnson and Brown, 2009). Moreover, heat-shock proteins (HSP70) are thought to induce transformation of promastigote stage of *Leishmania donovani* to its amastigote stage (Wiesgigl and Clos, 2001). Thioredoxin peroxidase, an antioxidant enzyme, is also identified in parasite-derived exosomes (Tzelos et al., 2016). It has been proposed that these vesicle-derived enzymes direct the immune system of host toward Th2 immune response, which is thought to be favorable for parasite development within the host (Robinson et al., 2010; Dalton et al., 2013). In addition to the proteins described above, MS spectra of tick-derived EVs were also analyzed for the presence of host proteins within these EVs. These host proteins have been known to be present in saliva of tick species and parasite-derived EVs (Buck et al., 2014; Kim et al., 2016; Samoil et al., 2018). In recent studies, it has been proposed that the host proteins like fibrinogen, serum albumin, and serotransferrin are likely associated with the events toward tick feeding (Kim et al., 2016). Therefore, presence of such proteins within tick-derived EVs clearly implicates these structures in host-parasite communication processes. However, their role in tick EVs requires further investigation.

Moreover, the proteome of *H. longicornis* tick saliva-derived EVs showed similarity with proteins identified from tick saliva (Supplementary Table 6). Proteins such as enolase, histones, heat shock proteins, lipocalin, thioredoxin, and vitellogenin have been identified within saliva of *Ornithodoros moubata, Ixodes scapularis, Rhipicephalus sanguineus, Amblyomma americanum, Dermacentor andersoni*, and *Haemaphysalis longicornis*. (Díaz-MartÍN et al., 2013; Oliveira et al., 2013; Mudenda et al., 2014; Tirloni et al., 2015; Ren et al., 2019). Due to the similarty in proteomes, it has been proposed that ticks could use these nano-sized vesicles to produce saliva (Díaz-MartÍN et al., 2013). Therefore, the findings of current study gave further confirmaton to assumptions derived in previous studies.

Gene Ontology (GO) database determines the functional annotation of gene products with a vocabulary of ontological terms describing their biological processes, molecular functions, and cellular components of the cell (Ashburner et al., 2000; Consortium, 2004; Thomas, 2017). GO data revealed that the possible outcomes of vesicular proteins were hydrolysis coupled proton transport, energy coupled proton transmembrane transport, and detoxification. “Biological process” indicated role of proteins in proton transport as well as in removal of harmful toxins accumulated within the body. In “cell component” category, highly enriched category was found to be “cell.” This analysis revealed that major biological as well as metabolic functions occur within cell. “Molecular function” analysis indicated that proteins were mainly involved in binding and catalytic activity. However, other significant functions associated with proteins were the transfer of ions across the membranes as well as inhibition of oxidation. KEGG pathways analysis showed that proteins regulated several pathways associated with ribosome biosynthesis (ribosome), transport of RNA species (RNA transport pathway), and regulation of oxidative pathway as well as generation of ATPs (oxidative phosphorylation). Bioinformatics analysis of vesicular proteins revealed that proteins are associated with certain biological as well as molecular functions which may be beneficial for reproduction and survival of ticks.

To the best of our knowledge, we report for the first time that the tick *H. longicornis* is also capable of secreting exosome-like vesicles. However, some limitations have been associated with the present study. This study deals with ticks, and saliva is the main excretory source of ticks, however, it is quite difficult to collect enough amount of saliva to be used separately for all the standard techniques. In addition, EVs isolated from saliva were just enough to be used for SDS-PAGE, electron microscopy, BCA, Western blot, and LC-MS/MS. Therefore, characterization of exosomes by nano-particle tracking analysis is not provided. At this stage, we would like to present the interesting results at an early stage to other researchers. Further categorization of tick-derived EVs is required to improve knowledge about the proteins within EVs and their associated functions.

## Conclusions

In summary, the present study constitutes the first analysis of secretion of EVs from ticks. The present study indicates that EVs may be a useful pathway for ticks for the transfer of genetic materials to host cells, thereby helping ticks in modulating host immune responses. However, better understanding of how EVs increase tick attachment to host skin as well as modulation of host immune responses will be helpful in pathogenesis and development of therapeutics and vaccine against ticks.

## Data Availability Statement

The datasets generated for this study can be found in the ProteomeXchange (accession number PXD020300).

## Ethics Statement

The animal study was reviewed and approved by Institutional Animal Care and Use Committee of Shanghai Veterinary Research Institute.

## Author Contributions

JZ conceived and designed the experiments. MN performed the experiments. HZ, JC, and YZ completed the data analysis. MM, IH, MH, and ZH contributed reagents, materials, analysis, and tools. JZ and MN wrote the paper. All authors read and approved the final manuscript.

## Conflict of Interest

The authors declare that the research was conducted in the absence of any commercial or financial relationships that could be construed as a potential conflict of interest.

## Supplementary Material

Supplementary Figure 1 Schematic representation of steps followed to purify tick EVs.

Supplementary Figure 2 Histograms representing significantly enriched (*P* ≤ 0.05) biological processes **(A)**, cellular components **(B)** and molecular functions **(C)**.

Supplementary Table 1 Protein content of tick-derived EVs identified using MASCOT search engine.

Supplementary Table 2 Host proteins identified in purified EVs.

Supplementary Table 3 Proteins present in supernatant of tick-derived EVs.

Supplementary Table 4 Details of Biological processes, cellular components and molecular functions.

Supplementary Table 5 KEGG Pathway analysis of proteins identified from tick-saliva derived EVs.

Supplementary Table 6 Common proteins between tick saliva and tick-derived EVs.
