# Supplementary figures and images for: Proteomic Analysis of Exosome-Like Vesicles Isolated From Saliva of the Tick Haemaphysalis longicornis
Source: Front Cell Infect Microbiol. 2020 Oct 22;10:542319. doi: 10.3389/fcimb.2020.542319 (PMC7642894; doi:10.3389/fcimb.2020.542319)

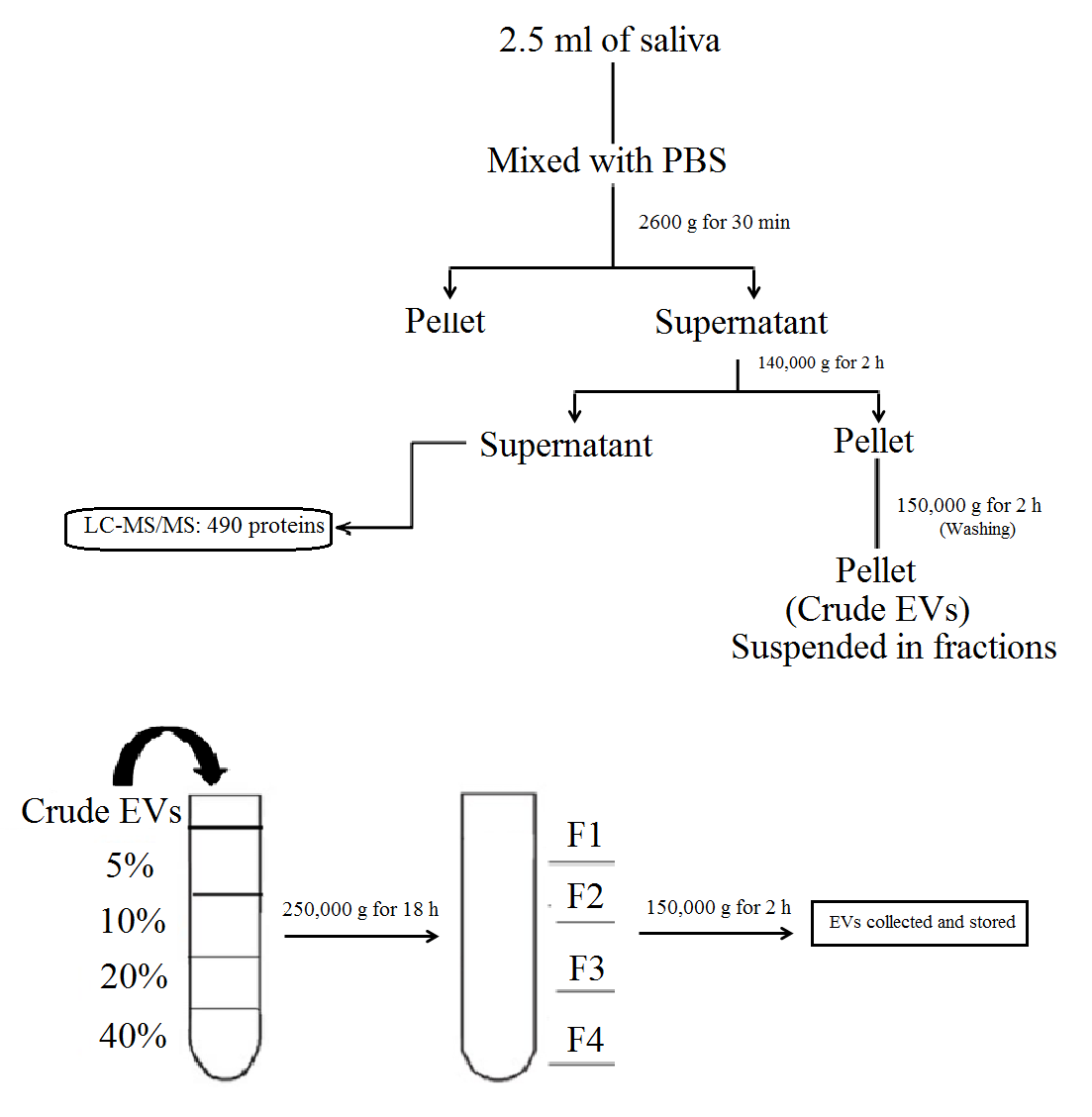

Supplement: Supplementary Figure 1 — Schematic representation of steps followed to purify tick EVs. [file Image_1.tif]

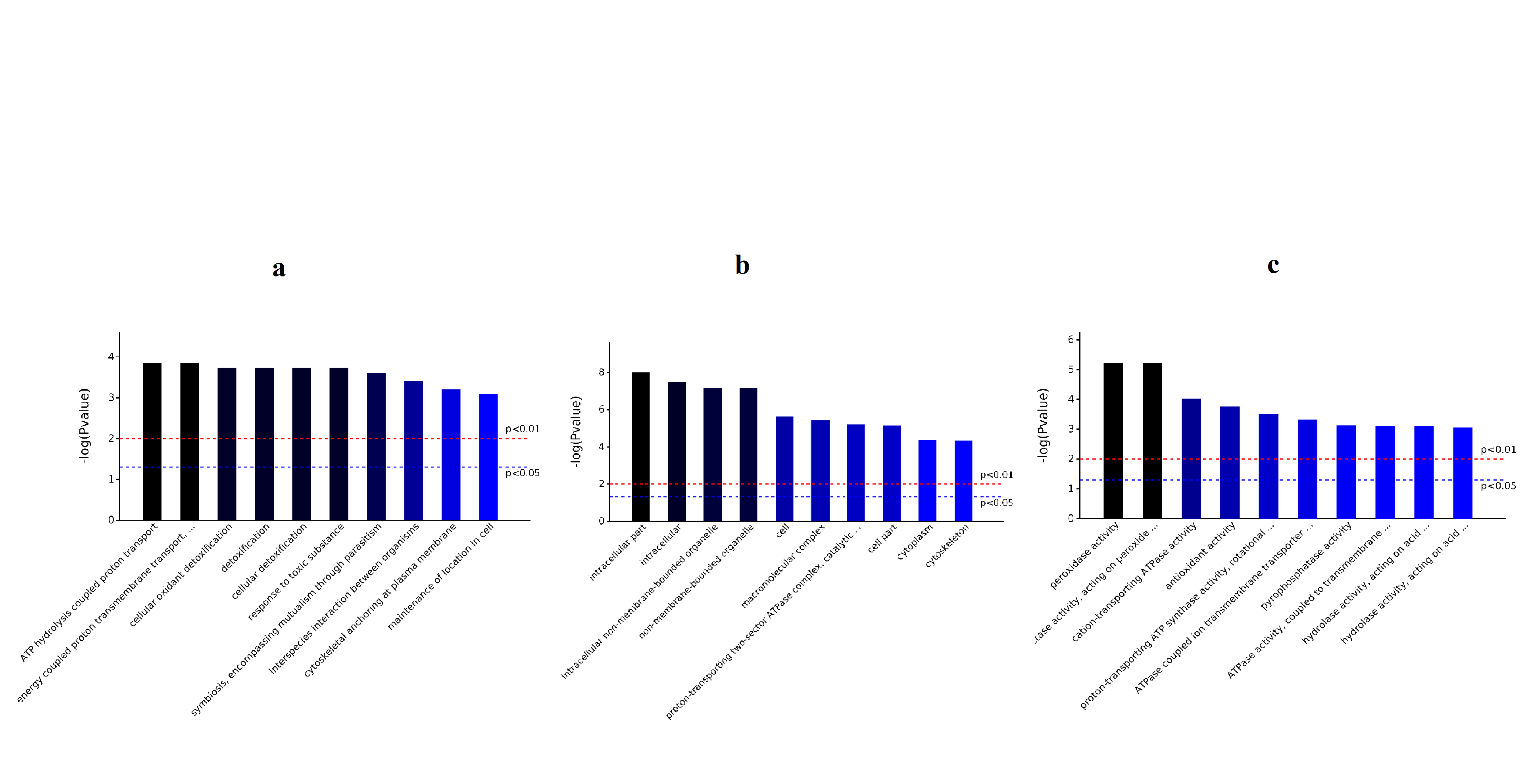

Supplement: Supplementary Figure 2 — Histograms representing significantly enriched (P ≤ 0.05) biological processes (A), cellular components (B) and molecular functions (C). [file Image_2.tif]
