# Supplementary material for: Proteomic Analysis of Exosome-Like Vesicles Isolated From Saliva of the Tick Haemaphysalis longicornis
Source: Front Cell Infect Microbiol. 2020 Oct 22;10:542319. doi: 10.3389/fcimb.2020.542319 (PMC7642894; doi:10.3389/fcimb.2020.542319)
Supplement: Supplementary Table 6 — Common proteins between tick saliva and tick-derived EVs. [file Table_6.docx]

**Supplementary Table 6. Common proteins between tick saliva and tick-derived EVs**

| **Accession no** | **Protein** | **Accession no** | **Protein** |
| --- | --- | --- | --- |
| A0A0K8R7B1 | **G**lutathione S-transferase | A0A131YNN3 | 15-hydroxyprostaglandin dehydrogenase |
| B7QCU5 | Sulfotransferase | G3MPT6 | Annexin |
| A0A293N4N6 | Catalase | A0A023FIY1 | Multifunctional chaperone |
| B7PDK3 | Cuticular protein | A0A023FJK7 | Heat shock protein |
| ACH88101 | Glyceraldehyde-3-phosphate dehydrogenase | A0A131YY80 | Heat shock protein 70 |
| A0A1E1X8M7 | ATP synthase subunit beta | A0A293N8J1 | Elongation factor 1-alpha |
| A0A1Z5KY25 | Malate dehydrogenase | A0A131YQ62 | Lipocalin |
| A0A224YHA0  ABQ96858  AGC13075  AEO34838  AEO34879  BAH02666.2 | **A**lpha-2-macroglobulin  Tropomyosin  **G**lutathione peroxidase  Histone H4  Histone H2A  Vitellogenin | A0A1D2AIB1  AEO32884  AEO34612  ADG86641  XP_002399568.1 | Zinc finger protein  Lipocalin  Enolase  Lysosomal acid phosphatase  Histones (H2A/H2B/H3/H4) |
